# Emergence of Multidrug-Resistant *Campylobacter jejuni* in a Common Variable Immunodeficiency Patient: Evolution of Resistance Under the Selective Antibiotic Pressure

**DOI:** 10.3390/tropicalmed10060165

**Published:** 2025-06-12

**Authors:** Tajana Juzbašić, Nataša Andrijašević, Ivana Ferenčak, Dragan Jurić, Silvija Šoprek, Vlatka Poje Janeš, Ljiljana Žmak, Arjana Tambić Andrašević, Ana Gverić Grginić

**Affiliations:** 1 Department of Microbiology, Croatian Institute of Public Health, 10000 Zagreb, Croatia; ivana.ferencak@hzjz.hr (I.F.); dragan.juric@hzjz.hr (D.J.); ljiljana.zmak@hzjz.hr (L.Ž.); ana.gveric-grginic@hzjz.hr (A.G.G.); 2 Department for Clinical Microbiology, University Hospital for Infectious Diseases, 10000 Zagreb, Croatia; natasa.andrijasevic@gmail.com (N.A.); silvija.soprek@gmail.com (S.Š.); atambic@bfm.hr (A.T.A.); 3 Department for Microbiology, Public Health Institute of Koprivnica Križevci County, 48000 Koprivnica, Croatia; jmpoje@yahoo.com; 4 School of Medicine, University of Zagreb, 10000 Zagreb, Croatia; 5 School of Dental Medicine, University of Zagreb, 10000 Zagreb, Croatia

**Keywords:** *Campylobacter jejuni*, recurrent infection, immunocompromised patients, CVID, multidrug resistance, whole genome sequencing

## Abstract

*Campylobacter jejuni* is a leading cause of bacterial gastroenteritis worldwide which usually presents as mild, and self-limiting disease in immunocompetent individuals. However, in immunocompromised patients, such as those with common variable immunodeficiency, *C. jejuni* can cause severe recurrent infections requiring antibiotic treatment. Our study reports a case of a 37-year-old male patient with CVID, who had multiple episodes of *C. jejuni* intestinal infections over a 3.5-year period. A total of 27 stool samples were collected and analyzed between December 2020 and July 2024 during acute febrile diarrheal episodes, with *C. jejuni* isolated in 15 samples. Antimicrobial susceptibility testing (AST) during the course of the disease revealed three different antimicrobial resistance profiles including multi-drug-resistant phenotype. Whole genome sequencing was performed on three representative isolates, all identified as MLST type 367, ST-257 complex, with minimal genetic divergence, indicating a clonal origin. Genes and point mutations conferring resistance to macrolides, fluoroquinolones, beta-lactams, and tetracycline were identified in different *C. jejuni* isolates, along with key virulence factors linked to adherence, invasion, motility, and immune evasion. The genetic analysis of macrolide phenotypic resistance revealed different resistance mechanisms. Genotypic and phenotypic analyses of the same *C. jejuni* clone from single patient, and identified multidrug resistance pattern, present the first documented case of in vivo resistance development of *C. jejuni* in Croatia. This case highlights the role of prolonged antibiotic pressure in driving resistance evolution and underscores the need for careful antimicrobial stewardship and genomic monitoring in immunocompromised patients. Further research is needed to correlate phenotypic resistance with genetic determinants in *Campylobacter* spp.

## 1. Introduction

*Campylobacter* (*C*) species (spp.), in particular *Campylobacter jejuni* and *Campylobacter coli*, are the major cause of bacterial gastroenteritis worldwide. Since 2005 in the European Union (EU) campylobacteriosis is the most reported zoonosis in humans [1,2,3]. It is mostly associated with the consumption of undercooked poultry, raw milk, and contaminated water. Campylobacteriosis in healthy individuals usually presents as a mild, self-limited disease, lasting five to seven days, while immunocompromised patients are at risk for severe, prolonged and recurrent infections that require antimicrobial treatment, which can contribute to the development of multidrug resistant (MDR) *Campylobacter* strains and treatment failure [4,5,6,7,8].

If *Campylobacter* spp. infection requires treatment, macrolides, and fluoroquinolones are the preferred first-line antibiotics [5]. Although tetracyclines are considered an alternative treatment option, they are infrequently used in clinical practice. In cases of severe or recurrent infections, multiple courses of antibiotics may be required, including the use of aminoglycosides or even carbapenems [4,9,10,11]. Fluoroquinolone-antimicrobial resistance among human *C. jejuni* isolates rapidly increases. Despite the evidence of stable resistance rate of *C. jejuni* to macrolides in many countries, recently some countries report an increase in erythromycin resistance levels. The widespread use of fluoroquinolones and macrolides in clinical treatments, and as growth promoters in food-producing animals, created the selective pressure for the emergence of fluoroquinolone-resistant and macrolide-resistant *Campylobacter* strains [12]. Due to the worldwide emergence of strains with a high level of resistance to fluoroquinolones, the World Health Organization (WHO) classifies *Campylobacter* spp. as a high-priority pathogen and includes it on the list of bacteria for which new antibiotics are urgently needed [13]. Therefore, campylobacteriosis requires constant active surveillance and diagnostic methods that can ensure adequate response to this emerging pathogen.

The main bacterial resistance mechanisms to fluoroquinolones, macrolides and tetracyclines in *Campylobacter* spp. are related to altered targets and efflux pumps. Fluoroquinolone resistance is chromosomally encoded and mostly mediated by point mutations in the quinolone resistance-determining region (QRDR) of DNA gyrase subunit A (*gyrA*). The most frequently reported mutation is the C257T change in the *gyrA* gene, which leads to the T86I substitution in the DNA gyrase and contributes to high level resistance to fluoroquinolones [14]. Other, less commonly reported mutations, include T86K, D90N and A70T, which in comparison to the T86I, rarely contribute to high level resistance to fluoroquinolones [14,15]. Macrolide resistance is mainly associated with target modification due to a point mutation in the genes for 23S rRNA and/or ribosome proteins L4 and L22 [14]. The most frequently reported mutations in *Campylobacter* spp. are A2075G, A2074C and A2074G that are found to confer a high-level resistance to macrolide antibiotics (for example erythromycin minimal inhibitory concentrations (MIC) > 128 μg/mL) while modifications of L4 and L22 contribute to low-level macrolide resistance [14,16,17]. Tetracycline resistance is mainly associated with the acquired *tet(O)* gene, encoding a ribosomal protection protein (RPR) [14,18].

Multidrug efflux pump, *cmeABC*, contributes to resistance to fluoroquinolones, macrolides and tetracyclines by expelling them from the bacterial cell via an energy dependent mechanism and thus reducing their intracellular concentrations [14].

The whole genome sequencing (WGS) has become a powerful and achievable tool allowing affordable, real-time, large-scale analysis of foodborne pathogens. This enables studying genetic variations that contribute to infection dynamics, virulence, and antimicrobial resistance [19]. This is especially important in infections that tend to persist in immunocompromised patients where pathogenic bacteria are subjected to prolonged environmental pressure from extended antibiotic or immunotherapy treatments.

WGS enables comprehensive profiling of resistance-associated genes, single nucleotide polymorphisms (SNPs), and mobile genetic elements, allowing for precise characterization of multidrug-resistant strains. Also, WGS allows for the identification and analysis of virulence genes that may influence a pathogen’s ability to colonize the host, evade immune responses, or persist in the host environment [20]. The relatedness of strains and the spread of specific lineages can be assessed by analyzing genomes, which provide insights into how specific *Campylobacter* strains evolve and adapt within a host over time [21]. By comparing WGS-derived and phenotypic antimicrobial resistance profile of *C. jejuni*, studies have shown that WGS is highly concordant with phenotypic antimicrobial susceptibility testing (AST) and therefore can be a good tool for predicting antimicrobial resistance (AMR) phenotypes for surveillance purposes [22,23].

This case report describes a patient with common variable immunodeficiency (CVID) who experienced multiple episodes of severe acute exacerbations of chronic colitis, accompanied with the formation of microabscesses in the colon, and persistent *C. jejuni* isolation in stool samples. CVID is a rare immunological disorder, with an estimated frequency of approximately 1 in 20,000 people. It is characterized by decreased serum levels of Immunoglobulin (Ig) G antibodies, in combination with IgA and/or IgM produced against various antigens. Hypogammaglobulinemia leads to compromised local immune response in the intestinal mucosa, which is a pathophysiological basis for recurrent infections caused by *Campylobacter* spp. [24]. Our research aimed to identify genetic characteristics of *C. jejuni* strains isolated in subsequent diarrheal episodes that followed different antibiotic treatment. These strains exhibit different antimicrobial susceptibility patterns. An additional aim to define was if it was a single, persistent sequence type that developed different AMR profiles over time under the pressure of antibiotics or it was persistent intestinal mucosa colonization with possible in vivo biofilm formation and occasional shedding off novel sessile genotypes.

## 2. Case Presentation

The patient, diagnosed with CVID in 2010, experienced the first manifestation of gastrointestinal symptoms in 2019. The detailed clinical course of the disease with the symptoms, and treatments is presented in Table 1. Between the dates in the table, the patient experienced short-term clinical improvements.

He was on continuous Ig replacement therapy from 2019 to 2024, initially receiving 80 g/800 mL monthly, increased to 100 g/1000 mL in 2020. Episodes of toxin-producing *C. difficile* colitis (2020, 2022) and repeated *C. jejuni* intestinal infections (from 2020 to 2024) complicated course of the disease, particularly following the emergence of multidrug-resistant strains in 2024. Ig therapy dosage remained stable since 2020, and no significant modifications were made aside from transient antibiotic pauses.

Despite initial responsiveness to antibiotics, treatment efficacy diminished as resistance developed, culminating in the use of broad-spectrum agents (meropenem, gentamicin) and consideration of fecal microbiota transplantation.

## 3. Materials and Methods

### 3.1. Sample Collection and Processing

#### 3.1.1. Sample Collection and Preparation

A total of 27 stool samples were collected and analyzed between December 2020 and July 2024 during the patient’s treatment and recurrent episodes of colitis. Fifteen samples were processed at the Public Health Institute of Koprivnica-Križevci County (PHIKC), eight at the University Hospital for Infectious Diseases Zagreb (UHID), and four at the University Hospital Centre Zagreb (UHC). Stool samples were stored at 4 °C if not processed immediately, and all samples were analyzed within 24 h of collection.

#### 3.1.2. Bacteriological Cultivation and Identification

All stool samples were cultured on campylobacter selective media: in PHIKC on Campylosel agar plates (bioMerieux, Marcy-l’Étoile, France), in UHID on CASA plates (bioMerieux, Marcy-l’Étoile, France), and in UHC on Columbia Skirrow plates (Oxoid, Basingstoke, Hampshire, UK). The cultures were incubated under microaerobic conditions at 42 °C for 48 h using CampyGen 2.5 L gas-generating kits (Thermo Fisher Scientific, Oxoid Limited, Hants, UK). After incubation, colonies were identified using classical biochemical tests (catalase, oxidase, hippurate hydrolysis test) and confirmed using matrix-assisted laser desorption/ionization time-of-flight mass spectrometry (MALDI-TOF) with the VITEK^®^ MS system (bioMérieux, Marcy-l’Étoile, France).

#### 3.1.3. Antimicrobial Susceptibility Testing (AST)

AST was performed on thirteen *C. jejuni* isolates following the European Committee on Antimicrobial Susceptibility Testing (EUCAST) guidelines [25]. The disk diffusion method was used to test susceptibility to erythromycin, ciprofloxacin, tetracycline, and gentamicin, while the gradient test was employed for assessing meropenem susceptibility. To meet the quality standards for susceptibility testing by the disk diffusion method, the *C. jejuni* ATCC 33560 reference strain was used.

Interpretation for erythromycin, ciprofloxacin, and tetracycline, was conducted according to EUCAST clinical breakpoints [25] while for gentamicin and meropenem was done according to EUCAST protocol for harmonized monitoring of antimicrobial resistance in *Salmonella* and *Campylobacter* and interpreted as susceptible (wild-type) or resistant (non-wild-type with potential resistance mechanisms) using epidemiological cut-off values (ECOFFs) [26].

From thirteen isolates with AST results, three isolates of interest were sent for conformational AST and molecular analysis to the Croatian Institute of Public Health (CIPH).

### 3.2. Genomics

#### 3.2.1. Library Preparation and Sequencing

DNA was extracted from the bacterial isolate using the NucleoSpin Tissue kit (Macherey-Nagel, Dueren, Germany), following the manufacturer’s instructions, which included a three-hour digestion with proteinase K at 56 °C. The WGS library with 2 × 150-bp reads, was prepared with the Illumina DNA prep kit and sequenced on the Illumina Nextseq 550 instrument (Illumina, San Diego, CA, USA). Quality control of the reads was performed with FastQC v0.12.1 [27] followed by trimming using Fastp v0.23.4 [28]. De novo genome assembly was carried out with the SPAdes genome assembler v3.10.1 under default settings and the “careful” option [29]. The assembled genome was further assessed for quality and G + C content using Quast software v5.2.0 [30]. Acceptable quality control (QC) parameters were maintained throughout the analytical procedure, and the data for this study have been deposited in the European Nucleotide Archive (ENA).

#### 3.2.2. Bioinformatic Tools

To detect the presence of *C. jejuni*/*coli* and for species confirmation of all samples, Kraken2 v2.1.2 was initially used with the minikraken2_v2_8GB_201904_UPDATE database [31]. Although Kraken2 predicted *C. jejuni*/*coli*, further analysis was conducted to achieve precise species clarification. Mash v2.3 was used to screen de novo assembled genomes against a custom sketch, which included reference genomes for *C. coli* (NZ_CP046317.1) and *C. jejuni* (NC_002163.1). This additional step allowed for accurate species differentiation, enhancing the reliability of the results [32]. We determined the similarity of the three *C. jejuni* strains with the dRep software tool v3.4.5 for genome comparison and clustering [33]. This analysis calculates the essential metrics for assessing genomic relatedness: MinHash Alignment for SHort reads (MASH) distances and Average Nucleotide Identity (ANI) values. DNA-DNA hybridization (DDH), which measures the degree of genetic similarity between organisms, was calculated using the Genome-to-Genome Distance Calculator v3.0 [34].

The Multi-Locus Sequence Type (MLST) and the clonal complex (CC) were determined using mlst v2.23.0 [35] and an online tool from https://pubmlst.org/ (accessed online on 25 March 2025) [36]. The core genome multi-locus sequence typing (cgMLST) that is used for high-resolution bacterial strain typing and evolutionary analysis by comparing genetic variations across the core genome is performed with the SeqSphere + software v10.5 (Ridom Gmbh, Münster, Germany). The antibiotic resistance genetic mechanisms were identified using RGI 6.0.3 software which scans the de novo genome assemblies against the CARD database 3.3.0 [37]. The analysis was conducted with the criteria set to select perfect, strict and loose hits, but with ≥95% identity of loose hits to strict which are stringent enough to ensure reliable detection and confirmation of the presence or absence of resistance genes.

The online tool Plasmid Finder v2.1 with the database v2023-01-18 was used to find possible plasmids [38,39]. To find genes that encode virulence factors Abricate v1.0.1 that scans VFDB databases was used [40,41].

## 4. Results

### 4.1. Phenotypic Results

#### 4.1.1. Species Identification/Confirmation

From December 2020 to July 2024, 27 stool samples were analyzed, of which 15 were positive for *C. jejuni*, and 12 were negative.

#### 4.1.2. Antimicrobial Susceptibility Testing

**Figure 1:**
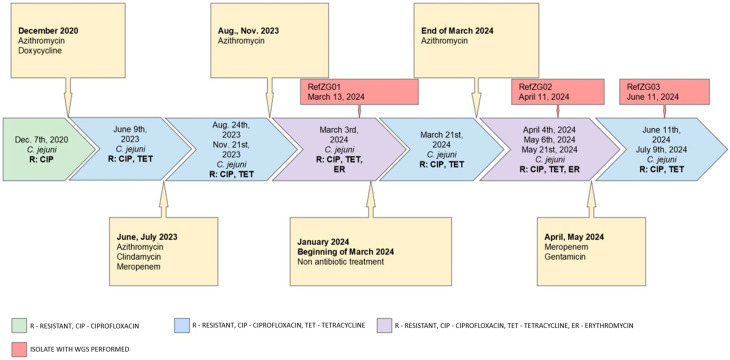
The timeline of isolates with their resistance profile.

Out of fifteen positive cultures, AST was successfully performed on thirteen *C. jejuni* isolates (KC01–KC06 and ZG01–ZG07). Two isolates were excluded due to failed subcultivation. Three distinct AST profiles were identified, with two alternating during the final episodes of *C. jejuni* colitis. The timeline of the isolated trains and their resistance profile is presented in Figure 1.

Four isolates collected between March and May 2024, exhibited an MDR phenotype, including KC05, KC06, ZG02, and ZG03, respectively. Subsequently, an isolated ZG04 showed restored susceptibility to macrolides. Identification and susceptibility profiles were confirmed by the national reference laboratory for selected isolates: KC05 (RefZG01), KC06 (RefZG02), and ZG04 (RefZG03). Detailed AST results of the samples analyzed are reported in Table 2.

### 4.2. WGS Results

#### 4.2.1. Species Identification/Confirmation

All three sequences were matched to *C. jejuni* subspecies *jejuni* NCBI: txid1248402, with distances of less than 0.008. The genomic similarity metrics between isolates is shown in Table 3.

All three isolates were MLST type 367, ST-257 complex and cgMLST for performing evolutionary analysis gave results presented in Table 4.

#### 4.2.2. Antimicrobial Resistance Genomic Profile

The genetic resistance profile confirmed the presence of MDR *C. jejuni*. The analysis of isolate RefZG01 revealed the presence of *bla_OXA_-461* gene from OXA-61 family class D beta-lactamase that harbors beta-lactam resistance, tetracycline resistance ribosomal protection protein-*tet(O)* gene. Also, distinctive point mutations were found on 23S ribosomal RNA that are responsible for resistance to macrolides (*23S_A2074C*), on DNA gyrase subunit A that confers resistance to quinolones (*gyrA_T86I*), and on the *bla_OXA_-61* promoter region, giving resistance to beta-lactams (*bla_OXA_-61_G-57T*) [42]. All isolates have genes for the *cmeABC* multidrug efflux system which is responsible for expelling the active substances out of the bacteria cell and thus reducing their intracellular concentration.

Isolates RefZG02 and RefZG03 have the same genetic resistance profile that differs from RefZG01 in that they have not got *23S_A2074C* mutation and thus should be sensitive to macrolides. The acquired antimicrobial resistance genes and point mutations are presented in Table 5.

#### 4.2.3. Virulence Genes

All three sequenced isolates showed identical virulence profiles when their sequence data were run against VFDB. Overall, 61 genes encoding adherence factors (5), invasion (2), motility (51), exotoxin (3), and immune modulation (1) were determined (Table 6).

## 5. Discussion

In this paper we presented a patient diagnosed with CVID who experienced repeated episodes of severe acute colitis and persistent isolation of *C. jejuni*. Such frequent episodes of campylobacteriosis occur in patients with underlying CVID, and are often present in severe forms [24]. As Janssen et al. reported in their study, an effective immune system (innate, cell-mediated, and humoral) is crucial for the host defense against *Campylobacter* spp. infection. Cellular immunity mechanisms involving decreased levels of CD4+ T-cell, CD8+ T-cell, B-cell, and NK-cell production are well known established risk factors [6,43]. An additional protective mechanism is the acidity of the stomach, and it is crucial in early stages of infection. Its impairment due to the pharmacological effect of gastritis treatment can promote infection by *Campylobacter* spp. [43].

Low or undetectable levels of IgA were recognized in different studies as risk factors for chronic infections caused by *Campylobacter* spp. Our patient was constantly receiving a regular application of Ig therapy, in the nine-year period, but he never reached the preferred reference levels of IgA, IgM, and IgG, as presumed for healthy individuals [44].

Successive isolation of *Campylobacter* spp. strains with different phenotypic antimicrobial susceptibility characteristics have raised several questions. The most important one was whether it involved the same clone colonizing the intestinal mucosa and inducing sporadic acute exacerbations of colitis or infections with novel strains. Chronic *Campylobacter* spp. intestinal carriage, recurrent infections, and the development of severe clinical manifestations are possible complications during CVID. Reported cases of chronic *Campylobacter* infection in patients with hypogammaglobulinemia, range from several months to six years with additional reports of potential cases lasting for 17 and 25 years [6].

Genomic relatedness confirmed by cgMLST indicates the origin of all three sequenced isolates from within the same genetic clone. We used cut-offs presented in a recent study on isolates from recurring infections [6,45]. These findings confirm clonal expansion and strongly suggest a persistent infection with the same strain rather than repeated reinfections from external sources. Despite the phenotypic variation in antibiotic susceptibility, the low genomic divergence in core genes points to in vivo in-host evolution rather than the introduction of new strains. This observation underscores the role of selective pressure in shaping resistance within a single clonal background.

The strain’s genetic capacity for infection has been demonstrated by the identified genes associated with virulence mechanisms and pathogenesis, including genes that facilitate disease development and those that promote extended bacterial survival and host colonization. As shown in Table 6, all three sequenced strains had the same genetic virulence profile with predominance of genes encoding flagellar motility (83.6%, 51/61). The prevalence of motility-associated genes suggests a significant capacity for mucosal colonization.

Aside from abundant motility genes, genetic virulence patterns included genes associated with the initial phase of invasion, particularly adhesion, specifically *cadF* and *JlpA*. Proteins encoded by these genes are crucial in illness development, since *Campylobacter* spp. infiltrates the epithelial cells by adhering to fibronectin via these proteins. The genes *caiB* and *caiC* have been linked to intracellular invasion, the survival of *Campylobacter* spp. in Campylobacter-containing vacuoles (CCV), and persistent infections within epithelial cells. Recurrent pathohistological findings in colon biopsy specimens taken during colonoscopies showed the presence of microabscesses, suggesting the potential for a protracted infection. The formation of these microabscesses may be associated with the presence of genes encoding cytolethal distending toxin which contributes to inflammation and the formation of microabscesses in the mucosa [6,46]. These toxins stimulate IL-8 release and attract neutrophils, hence enhancing the inflammatory response [47].

The persistence of the uniform virulence factors across phenotypically different but clonally related isolates suggests that resistance developed without loss of pathogenicity. This presents a serious clinical concern in immunocompromised patients, where such strains can sustain colonization and resist treatment.

It was also important to address the question of how and through which genetic mechanism antimicrobial resistance developed. The emergence of MDR isolates during later stages of infection is clinically significant, suggesting in vivo evolution of resistance that was likely driven by repeated antibiotic exposure over time. While this shift in resistance may indicate selection of a less-resistant subpopulation, further genomic analysis was necessary to distinguish between reversion, mixed colonization, or re-infection. Fluoroquinolone resistance was consistently detected in all isolates tested, indicating that this resistance trait was stable and not subject to variation during the observed period. Resistance to tetracycline was present in most isolates and often co-occurred with macrolide resistance in MDR phenotypes.

These results highlight a dynamic resistance landscape, with significant clinical implications. The detection of resistance shifts, particularly the development of MDR profiles, emphasizes the need for cautious and targeted antimicrobial use, especially in immunocompromised patients where chronic colonization can drive resistance evolution.

As was previously described, the main bacterial resistance mechanisms to fluoroquinolones, macrolides, and tetracyclines in *Campylobacter* spp. are related to altered targets and efflux pumps [14,15,16,17,18,42]. In this study, the genetic determinants of AMR to the mentioned classes of antimicrobials were analyzed through WGS sequencing in three *C. jejuni* strains (RefZG01, RefZG02 and RefZG03). The genetic resistance mechanisms were found as follows: *gyrA_T86I* point mutation (RefZG01, RefZG02 and RefZG03) contributing to a high-level resistance to ciprofloxacin, *23S_A2074C* point mutation (RefZG01) contributing to a high-level resistance to erythromycin, *tet(O)* gene (RefZG01, RefZG02, and RefZG03) contributing to resistance to tetracycline, as well as *cmeABC*, genes for multidrug efflux pump (RefZG01, RefZG02 and RefZG03) contributing to resistance to fluoroquinolones, macrolides and tetracyclines. This AMR gene profile backs up phenotypic findings. The presence of fluoroquinolone resistance in all three isolates attributed to *gyrA* mutation, according to Alfredson et al., is the most common worldwide, except Australia, while induction of fluoroquinolone resistance during treatment is also well recognized and has been reported to arise under selective pressure but not to withdraw [42].

The presence of macrolide resistance attributed to *23S_A2074C* mutation in RefZG01, but was not confirmed in RefZG02, along with detection of *cmeABC* multidrug efflux system genes in all three isolates, raises the possibility of induced gene expression in RefZG02 that lacks distinct genes or point mutations responsible for macrolide resistance. Macrolide resistance in RefZG01 and RefZG02 is a possible result of selective pressure imposed by azithromycin therapies (point mutation and induction of gene expression of multidrug efflux pump). The RefZG02 and RefZg03 either lost or possibly did not acquire the *23S_A2074C* mutation under the therapy with macrolide antibiotic. However, RefZG02 expresses phenotypic resistance, possibly due to the induction of an efflux mechanism under macrolide antimicrobial pressure. When treatment with azithromycin is discontinued, gene expression is suppressed resulting in the restoration of sensitivity. Even though WGS is a good tool for predicting AMR phenotypes for surveillance purposes, in certain cases gene expression analyses are necessary to confirm the connection between genotype and expressed phenotype [22,23]. These types of analyses were not conducted in this study, but future research could focus on exploring this aspect further as without the proven induction of genomic expression we cannot exclude the possibility that the mutation was present unevenly within the bacterial population, and therefore different results were produced depending upon the colony chosen for phenotypic testing.

This paper presents the first documented case of in vivo evolution of *Campylobacter jejuni* MDR in Croatia. Clinical outcomes, such as this, lead to difficult-to-treat and frequently recurring infections, illustrate the complexity of managing chronic infections in immunocompromised patients and raise the question of treatment options in the future. In recent years, hematopoietic stem cell transplantation has emerged as a potential therapeutic option as well as fecal transplantation [44]. Further research is needed to evaluate their efficacy in this context and to develop effective approaches for managing antimicrobial resistance in vulnerable patient populations.

## 6. Conclusions

Repeated courses of different antibiotic regimens resulted in the development of an MDR strain, driven by the selective pressure from antibiotic treatments. Notably, this case reports the first documented case of in vivo resistance development in Croatia and highlights the potential for in vivo development of antibiotic resistance in *C. jejuni* because of prolonged chronic infection and repeated exposure to various classes of antimicrobial agents. Our aim was to underscore the risk of resistance emergence during long-term antibiotic therapy in immunocompromised patients, where treatment often becomes increasingly complex. Importantly, a favorable clinical outcome depends not only on the appropriate selection of antimicrobial therapy, but also on the delicate balance between the host’s immune system and the pathogen. Future strategies should consider both the optimization of antimicrobial regimens and the modulation of host immune responses to more effectively counteract the development of antibiotic resistance.

## Figures and Tables

**Table 1:** Clinical course of the disease.

Date Range	Symptoms	Isolates	Therapy
2019	abdominal cramps, diarrhea, bloating and weight loss	*C. jejuni* negative	oral ciprofloxacin (CIP) (0.5 g twice daily) and subcutaneous IG therapy (80 g/800 mL per month)
01/2020	worsening GI symptoms (diarrhea, abdominal cramps and fever)	Toxin-producing *C. difficile*	oral vancomycin (0.125 g four times daily), metronidazole (0.4 g three times daily), corticosteroids (0.024 g/day), and mesalazine (1 g three times daily), along with an increased Ig dose to 100 g/1000 mL per month
12/2020	worsening GI symptoms	*C. jejuni*	oral azithromycin (0.5 g once daily)
2021–2022	intermittent diarrhea	*C. jejuni* negative	no reported treatment
12/2022	intermittent diarrhea	Toxin-producing *C. difficile*	oral vancomycin (0.125 g four times daily)
02/2023	diarrhea (25 watery stools/day), fever of 38 °C, ICU admission for treatment of malabsorption, electrolyte imbalance, and subsequent pneumonia	*C. jejuni* negative	oral vancomycin (0.125 g four times daily), levofloxacin for pneumonia
06/2023	diarrhea, fever	*C. jejuni* R: CIP ^1^, TET ^2^	oral azithromycin (0.5 g once daily) followed by oral clindamycin (0.3 g four times daily). Hospitalization with fever and treated with intravenous (IV) meropenem (1 g three times daily)
08/2023	diarrhea	*C. jejuni* R: CIP, TET	oral azithromycin (0.5 g once daily)
11/2023	diarrhea	*C. jejuni* R: CIP, TET	oral azithromycin (0.5 g once daily)
03/2024	diarrhea with 3–5 s stools per day, abdominal discomfort, and low-grade fever	*C. jejuni* R: CIP, TET, ER ^3^ *C. jejuni* R: CIP, TET	oral azithromycin (0.5 g once daily)
04–05/2024	persistent diarrhea	*C. jejuni* R: CIP, TET, ER	supportive therapy only
05/2024	fever, 15 diarrheal stools daily, and abdominal cramps	*C. jejuni* R: CIP, TET, ER *C. jejuni* R: CIP, TET	IV meropenem (1 g three times daily)- discontinued after 10 days due to leukopenia. IV gentamicin (0.24 g once daily) for four days, with prophylactic vancomycin.
06–07/2024	abdominal pain, spasms, and flatulence	*C. jejuni* R: CIP, TET	Supportive therapy, nutritional supplementation, probiotics

^1^ CIP—Ciprofloxacin. ^2^ TET—tetracycline. ^3^ ER—Erythromycin.

**Table 2:** The phenotypic results determined by the disk diffusion method (S—susceptible, R—resistant, WT—wild-type).

Antimicrobial Agent (Class)					
Isolate/ Ref. Isolate	Erythromycin (Macrolide)	Ciprofloxacin (Fluoroquinolone)	Tetracycline (Tetracycline)	Gentamycin (Aminoglycoside)	Meropenem (Beta-Lactam)
KC01	S	R	S	-	-
KC02	S	R	S	-	-
KC03	S	R	R	-	-
KC04	S	R	R	-	-
KC05/RefZG01	R/R	R/R	R/R	-/WT	-
ZG01	S	R	R	-	-
KC06/RefZg02	R/R	R/R	R/R	-/WT	
ZG02	R	R	R	WT	WT
ZG03	R	R	R	WT	WT
ZG04/RefZG03	S/S	R/R	R/R	-	-
ZG05	S	R	R	-	-
ZG06	S	R	R	-	-
ZG07	S	R	R	-	-

**Table 3:** Genomic similarity metrics: MASH distance, average nucleotide identity and DNA-DNA hybridization.

	RefZG01 vs RefZG02	RefZG01 vs RefZG03	RefZG02 vs RefZG03
MASH ^1^ distance	0.000024	0.000024	0.000024
ANI ^2^ (%)	99.9	99.9	99.9
DDH ^3^ (%)	100	99.9	99.9

^1^ MASH—MinHash alignment for SHort reads. ^2^ ANI—average nucleotide identity. ^3^ DDH—DNA-DNA hybridization.

**Table 4:** cgMLST genetic distance between isolates.

	RefZG01	RefZG02	RefZG03
RefZG01	0	9	9
RefZG02	9	0	3
RefZG03	9	3	0

**Table 5:** Genetic resistance profiles and point mutations.

Isolates	AMR Genes	Point Mutations Associated with AMR
RefZG01	*bla_OXA_-461*	*bla_OXA_-61_G-57T*
	*tet(O)*	*gyrA_T86I*
	*cmeA*, *cmeB*, *cmeC*, *cmeR*	*23S_A2074C*
RefZG02	*bla_OXA_-461*	*bla_OXA_-61_G-57T*
	*tet(O)* *cmeA*, *cmeB*, *cmeC*, *cmeR*	*gyrA_T86I*
RefZG03	*bla_OXA_-461*	*bla_OXA_-61_G-57T*
	*tet(O)* *cmeA*, *cmeB*, *cmeC*, *cmeR*	*gyrA_T86I*

**Table 6:** Virulence-related genes grouped by virulence category.

Isolate	Adherence Related Genes	Invasion Related Genes	Motility Related Genes	Exotoxin Related Genes	Immune Modulation Related Genes
RefZG01	*cadF*, *jlpA*, *pebA*, *porA*, *cj1279c*	*ciaB*, *ciaC*	*fla(C*, *-G*, *-A)*, *flg(B*, *-C*, *-D*, *-E*, *-G*, *-G2*, *-H*, *-I*, *-J*, *-K*, *-L*, *-M*, *-P*, *-Q*, *-R*, *-S)*, *flh(A*, *-B*, *-F*, *-G)*, *fli(A*, *-D*, *-E*, *-F*, *-G*, *-H*, *-I*, *-K*, *-L*, *-M*, *-N*, *-P*, *-Q*, *-R*, *-S*, *-W*, *-Y)* *pse(B*, *-C*, *-F)* *rpoN* *che(A*, *-V*, *-W*, *-Y)* *eptC* *Cj0371*, *Cj0883c*	*cdtA*, *cdtB*, *cdtC*	*Cj1135*
RefZG02	*cadF*, *jlpA*, *pebA*, *porA*, *cj1279c*	*ciaB*, *ciaC*	*fla(C*, *-G*, *-A)*, *flg(B*, *-C*, *-D*, *-E*, *-G*, *-G2*, *-H*, *-I*, *-J*, *-K*, *-L*, *-M*, *-P*, *-Q*, *-R*, *-S)*, *flh(A*, *-B*, *-F*, *-G)*, *fli(A*, *-D*, *-E*, *-F*, *-G*, *-H*, *-I*, *-K*, *-L*, *-M*, *-N*, *-P*, *-Q*, *-R*, *-S*, *-W*, *-Y)* *pse(B*, *-C*, *-F)* *rpoN* *che(A*, *-V*, *-W*, *-Y)* *eptC* *Cj0371*, *Cj0883c*	*cdtA*, *cdtB*, *cdtC*	*Cj1135*
RefZG03	*cadF*, *jlpA*, *pebA*, *porA*, *cj1279c*	*ciaB*, *ciaC*	*fla(C*, *-G*, *-A)*, *flg(B*, *-C*, *-D*, *-E*, *-G*, *-G2*, *-H*, *-I*, *-J*, *-K*, *-L*, *-M*, *-P*, *-Q*, *-R*, *-S)*, *flh(A*, *-B*, *-F*, *-G)*, *fli(A*, *-D*, *-E*, *-F*, *-G*, *-H*, *-I*, *-K*, *-L*, *-M*, *-N*, *-P*, *-Q*, *-R*, *-S*, *-W*, *-Y)* *pse(B*, *-C*, *-F)* *rpoN* *che(A*, *-V*, *-W*, *-Y)* *eptC* *Cj0371*, *Cj0883c*	*cdtA*, *cdtB*, *cdtC*	*Cj1135*

## Data Availability

The original data presented in the study are openly available in European Nucleotide Archive at https://www.ebi.ac.uk/ena/browser/view/PRJEB64612 (accessed on 25 March 2025). The experiment accession numbers are ERX13008384, ERX13008385, and ERX13008386.
